# Oxidative Stress, Inflammation, and Autophagy: Potential Targets of Mesenchymal Stem Cells-Based Therapies in Ischemic Stroke

**DOI:** 10.3389/fnins.2021.641157

**Published:** 2021-02-26

**Authors:** Jialin He, Jianyang Liu, Yan Huang, Xiangqi Tang, Han Xiao, Zhiping Hu

**Affiliations:** ^1^Department of Neurology, The Second Xiangya Hospital, Central South University, Changsha, China; ^2^National Health Commission Key Laboratory of Birth Defect for Research and Prevention, Hunan Provincial Maternal and Child Health Care Hospital, Changsha, China

**Keywords:** mesenchymal stem cell, cerebral ischemic injury, oxidative stress, inflammation, autophagy dysfunction, extracellular vesicles

## Abstract

Ischemic stroke is a leading cause of death worldwide; currently available treatment approaches for ischemic stroke are to restore blood flow, which reduce disability but are time limited. The interruption of blood flow in ischemic stroke contributes to intricate pathophysiological processes. Oxidative stress and inflammatory activity are two early events in the cascade of cerebral ischemic injury. These two factors are reciprocal causation and directly trigger the development of autophagy. Appropriate autophagy activity contributes to brain recovery by reducing oxidative stress and inflammatory activity, while autophagy dysfunction aggravates cerebral injury. Abundant evidence demonstrates the beneficial impact of mesenchymal stem cells (MSCs) and secretome on cerebral ischemic injury. MSCs reduce oxidative stress through suppressing reactive oxygen species (ROS) and reactive nitrogen species (RNS) generation and transferring healthy mitochondria to damaged cells. Meanwhile, MSCs exert anti-inflammation properties by the production of cytokines and extracellular vesicles, inhibiting proinflammatory cytokines and inflammatory cells activation, suppressing pyroptosis, and alleviating blood–brain barrier leakage. Additionally, MSCs regulation of autophagy imbalances gives rise to neuroprotection against cerebral ischemic injury. Altogether, MSCs have been a promising candidate for the treatment of ischemic stroke due to their pleiotropic effect.

## Introduction

Stroke is a devastating and debilitating medical condition in the world, consisting of ischemic stroke and hemorrhagic stroke. Ischemic stroke is an infarction of the brain, spinal cord, or retina, accounting for approximately 79% of all strokes globally (GBD 2017 Disease and Injury Incidence and Prevalence Collaborators, 2018). It is estimated that one in four adults will experience a stroke, and there are more than 80 million stroke survivors with varying degrees of disability that affect their quality of life worldwide (Campbell et al., 2019). Accordingly, it is imperative to search for effective therapeutic options in order to reduce the mortality and disability rate.

Currently available treatment approaches for ischemic stroke are to restore blood flow through intravenous thrombolysis (Anderson et al., 2019) and endovascular recanalization (Albers et al., 2018; Nogueira et al., 2018), which both reduce disability but are time bound. Stem-cell-based therapies are generally accepted as emerging paradigm in stroke (Boltze et al., 2019) and have gradually translated into clinical trials. Mesenchymal stem cells (MSCs) have been an appealing candidate for the treatment of ischemic stroke due to their easy accessibility, multidirectional differentiation potential, and immunomodulatory properties (Li et al., 2020b).

The blockage of blood flow during stroke results in complicated pathophysiological processes, which are comprised of oxidative stress, inflammation, breakdown of blood–brain barrier (BBB), calcium overload, excitotoxity, and autophagy dysfunction, contributing to neural disaster (George and Steinberg, 2015). The subsequent recanalization of blood flow always leads to secondary injury, namely, reperfusion injury (Granger and Kvietys, 2015). Oxidative stress and inflammatory activity are two early events in the cascade of cerebral ischemic injury, causing the disruption of numerous neural circuits (Rana and Singh, 2018; Chen et al., 2020a, b). Autophagy, as a scavenger, also activated upon cerebral ischemia and exerts a biphasic effect on ischemic stroke. Appropriate autophagy contributes to maintaining cerebral metabolism, while autophagy imbalance aggravates cerebral damage (Hu et al., 2015). Prior evidence has demonstrated the ability of MSCs to combat these pathophysiological processes in ischemic stroke. Therefore, in this review, we will outline the mechanism of MSCs in alleviating oxidative stress as well as inflammation and regulating autophagy during cerebral ischemia–reperfusion injury.

## Oxidative Stress, Inflammation, and Autophagy After Brain Ischemia

Plentiful data have implied that oxidative stress, neuroinflammation, and autophagy dysfunction worked together to damage cells after brain ischemia. Subsequently, we will inquire into the exact mechanisms and crosstalk among oxidative stress, inflammatory activity, and autophagy in ischemic stroke.

### Oxidative Stress

Oxidative stress is a key link in the cascade of cerebral ischemia–reperfusion injury. It is caused by the elevated production of reactive oxygen species (ROS) and reactive nitrogen species (RNS), which inhibit the normal function of lipids as well as proteins and induce DNA modification (Allen and Bayraktutan, 2009). ROS is a by-product of oxygen metabolism in mitochondria and composed of superoxide anions (O_2_^–^), hydrogen peroxide, hydroxyl radical, and hydroperoxyl radical. RNS mainly includes nitric oxide (NO) and peroxynitrite anion (ONOO^–^); the latter is formed by the rapid reaction of NO and O_2_^–^ (Kang and Pervaiz, 2012).

During cerebral ischemia, the mechanisms regarding the abnormal acceleration of ROS and RNS are basically as follows: First, hypoxia interrupts the oxidative phosphorylation process of the mitochondrial respiratory chain (MRC), leading to the depolarization of mitochondria and an increased level of O_2_^–^. Meanwhile, the acidic environment caused by hypoxia further accelerates the conversion of O_2_^–^ to other types of ROS (Saeed et al., 2007). Second, the N-methyl-D-aspartate (NMDA) receptors are activated by the elevated extracellular glutamate, contributing to an increase in calcium influx; the latter aggravates mitochondrial dysfunction and activates cellular proteases and lipases (Manzanero et al., 2013). Additionally, NMDA receptors also trigger the nitric oxide synthase (NOS), which catalyze L-arginine to produce NO, giving rise to an increase in ONOO^–^ production (Chen et al., 2013). Third, other oxidases, including xanthine oxidase and reduced nicotinamide adenine dinucleotide phosphate (NADPH) oxidase, are also responsible for the raised ROS level (Furuhashi, 2020). Fourth, endoplasmic reticulum stress and Golgi apparatus stress triggered by oxidative stress signals disrupt Ca^2+^ homeostasis and promote the advancement of oxidative stress (Jiang et al., 2011; Nakka et al., 2016).

Reactive oxygen species and RNS play a crucial biological role in the normal physiological processes. ROS is involved in cell signaling, immune defense, cell senescence, apoptosis, and the decomposition of toxic compounds (Bergendi et al., 1999), and NO participates in the regulation of neural signaling and immunological surveillance. However, in ischemic stroke, excessive ROS and RNS production has a detrimental impact on the neuron, glia cells, and vascular endothelial cells, such as swelling and necrosis of organelles, lipid peroxidation, protein denaturation, DNA modification as well as fragmentation, autophagy induction, and apoptosis (Allen and Bayraktutan, 2009).

The secretion of inflammatory molecules and the activation of inflammasomes can be directly triggered by the overwhelming production of ROS and RNS. Therefore, the attack of oxidative stress on nerve tissues is always accompanied by inflammatory cascade, both of which lead to apoptosis through tanglesome pathways involving p53 (Zhang et al., 2019a), p38 mitogen-activated protein kinase (MAPK) (Song and Ren, 2019), ERK1/2 (Wang et al., 2020a), and Keap1–Nrf2 pathway (Guo and Mo, 2020). Besides, ROS and RNS overproduction usually exacerbates the disruption of BBB on account of their ability to induce the vasodilatation and increase the permeability of vascular endothelial cells (Bao et al., 2018). More importantly, RNS-mediated caveolin-1 and matrix metalloproteinase (MMP) signaling pathways participate in the progression of neuroinflammation and disruption of BBB (Chen et al., 2018). Collectively, targeting the overproduction of ROS and RNS might provide satisfactory outcome in the treatment regarding ischemic stroke.

Additionally, since oxidative stress is a powerful trigger for cell death and relevant to the development of various diseases, including atherosclerosis, cardiovascular/cerebrovascular diseases, and psychiatric disorders, the clinical mirror of oxidative stress is under intensive investigation. Prior studies suggested the levels of oxidative stress and antioxidant system in serum as the predictor of the response to treatment or prognosis (Zitnanova et al., 2016; Hendouei et al., 2018; Darroudi et al., 2020). The reduction in antioxidant enzyme activity was found to be negatively correlated with clinical neurological soft signs of patients with psychiatric disorders (Raffa et al., 2012; Miljevic et al., 2018), and the prooxidative–antioxidative balance was identified as a well predictor of outcome in acute ischemic stroke (Kotur-Stevuljevic et al., 2015). Thus, therapeutic regimens that downregulate the levels of oxidants or upregulate antioxidants levels may contribute to clinically functional recovery (Chamorro et al., 2016; Elsner et al., 2021).

### Inflammatory Activity

Inflammatory activity is another pivotal cascade behavior following cerebral ischemia. Inflammatory activity is initially designed to help clear away damaged tissues and promote synapse reconstruction through the cytokines released by immune cells under physiological conditions, while continuous inflammatory activity following stroke may aggravate the catastrophes of nerve tissues (Jayaraj et al., 2019).

After cerebral ischemia, the brain-tissue-resident microglia first respond, activate, and aggregate to the infarction lesion, followed by the accumulation of peripheral-derived macrophages, neutrophils, dendritic cells (DCs), and lymphocytes in the peri-infarct region (Gelderblom et al., 2009; Iadecola and Anrather, 2011). These brain-resident microglia and blood-derived macrophages are able to release proinflammatory factors including tumor necrosis factor-α (TNF-α) and interleukin-1 (IL-1) and cell adhesion molecules and proteases, further propagating inflammatory activity and tissue damage (George and Steinberg, 2015; Jayaraj et al., 2019). Besides, microglia and macrophages also produce NADPH oxidase and inducible nitric oxidase synthase (iNOS) (Carbone et al., 2015; Zrzavy et al., 2018), giving rise to the overproduction of ROS and RNS and the advancement of oxidative stress.

It is well-known that microglia/macrophages can polarize to classic proinflammatory type (M1-like) and alternative protective type (M2-like) under diverse stimuli (Xiong et al., 2016; Ma et al., 2017). More precisely, microglia/macrophages polarize toward M1-like state under the stimulation of interferon (IFN), TNF-α, and Toll-like receptor 4 (TLR4) activation, while they polarize toward M2-like state by protective cytokines and cell debris. The function of M1 and M2 microglia/macrophages has been fully studied, where M1 microglia/macrophages produce proinflammatory molecules as previously mentioned, and M2 phenotype is responsible to suppress inflammation and promote tissue recovery (Guruswamy and ElAli, 2017; Kanazawa et al., 2017). During cerebral ischemia, macrophages/microglia are more inclined to polarize toward M1-like state in the ischemic penumbra region (Wang et al., 2018a), causing a cascade of inflammation. Consequently, the exploitation of approaches to promote the M2-like polarization of microglia/macrophages for attenuating the destructive effect of cerebral ischemia has become an interesting and meaningful topic and started to take effect (Xia et al., 2015; Qin et al., 2017; Lu et al., 2019; Zhao et al., 2019a, b).

In addition to macroglia/macrophages, astrocyte, peripheral-derived neutrophils, DCs, and lymphocytes are also involved in the development of inflammatory activity. Similar to microglia, astrocytes are also divided into neurotoxic and neuroprotective phenotypes, called classical and alternative activated astrocytes or A1 and A2 astrocytes, which exhibit proinflammatory and anti-inflammatory effects, respectively (Hong et al., 2020). A1 astrocytes are derived from resting astrocytes stimulated by the inflammatory factors including TNF-α, IL-1α, and C1q generated from activated microglia (Liddelow et al., 2017), and A1 astrocyte has been ascertained to be elevated after cerebral ischemia. A1 activated astrocyte not only delivery inflammatory mediators, such as TNF-α, IL-1, glial fibrillary acidic protein (GFAP), and matrix metalloproteases (MMPs), but also promote the formation of glial scar and disrupt the BBB (Jayaraj et al., 2019; Popa-Wagner et al., 2019). Meanwhile, the infiltrated neutrophils, DCs, and lymphocytes also promote proinflammatory pathways via secreting inflammatory factors and endothelial adhesion molecules (Weston et al., 2007; Anrather and Iadecola, 2016). Furthermore, dangerous-associated molecular patterns (DAMPs) released by the dying neurons and microglia in turn further motivate those immune cells and promote inflammatory tragedy. High-mobility group box 1 protein (HMGB1), as a star molecule of DAMPs, has been identified to participate in the inflammatory activity and aggravate brain injury in ischemic stroke (Liesz et al., 2015; Ye et al., 2019).

To date, pyroptosis, as a new definition of programed cell necrosis, have become therapeutic target of concern regarding inflammatory diseases (Jorgensen and Miao, 2015). Pyroptosis is manifested by the rapid cell lysis, resulting in the release of cell contents and the activation of intense inflammatory response (Shi et al., 2017). More concretely, pyroptosis begins with inflammasomes stimulated by diverse DAMPs, and the several celebrated inflammasomes contain NLRP1, NLRP3, NLRC4, ASC, and AIM2 (Poh et al., 2019; Yang et al., 2020b). Subsequently, the activated inflammasomes induce the maturation of caspase-1, interleukin-1β (IL-1β), and IL-18 (Wang et al., 2020b). The Gasdermin D (GSDMD) is the key substrate protein of caspase-1-mediated pyroptosis, where caspase-1 cleaves the linker between N- and C-terminals of GSDMD to block the autoinhibitory interactions between these two domains (Zhang et al., 2019b). It is well documented that inflammasome activation and pyroptosis indeed exist in neurons, microglia, and astrocytes following ischemic stroke (Xu et al., 2019; Ye et al., 2020; Zhang et al., 2020), worsening cerebral ischemic injury, while targeting pyroptotic components produces considerable neuroprotective benefit (Jiang et al., 2020; Yang et al., 2020b).

### Autophagy

Autophagy is a dynamic process of self-degradation of intracellular components including organelles and proteins and mediated by multiple lysosomal enzymes (Cecconi and Levine, 2008). Mammalian autophagy has been classified into three types: macroautophagy, microautophagy, and chaperone-mediated autophagy (CMA) (Sun et al., 2018). In general, macroautophagy is what we call autophagy. Autophagy is often activated by nutrient deficiency and metabolic stress and regulated by a complicated signaling network, which is essential for maintaining the homeostasis of intracellular environment (Ravanan et al., 2017).

Of note, autophagy-related signaling pathways in neurons, glia cells, and brain microvascular cells have been confirmed to be notably activated upon cerebral ischemia (Wang et al., 2018b), where neuronal autophagy was obviously early. Autophagy is induced by a variety of stimulating molecules after cerebral ischemia. First, the interruption of energy supply results in attenuated activation of the main inhibitor of autophagy, the mammalian target of rapamycin complex 1 (mTORC1), by nutrients, accompanied by the activation of AMP-activated protein kinase (AMPK), which both induce the enhancement of autophagy (Chong et al., 2013; Cespedes et al., 2019). Second, a large number of unfolded proteins produced by endoplasmic reticulum stress (Hou et al., 2019), excitotoxicity-mediated NMDA receptor activation, intracellular calcium overload, overproduction of ROS caused by mitochondrial dysfunction (Lee et al., 2012; Li et al., 2015b), and excessive RNS level (Feng et al., 2017) are all involved in the activation of autophagy. Third, evidence regarding the interplay of autophagy-inflammatory activity in ischemic stroke has also been unveiled (Mo et al., 2020), in which the activation of inflammation directly triggers the autophagy.

The regulated pathways of autophagy during cerebral ischemia affected by the above factors have been studied widely, which overlap with inflammation and oxidative stress and mainly involve mTOR signaling pathway (classic type I PI3K/Akt-mTOR and AMPK-mTOR pathway) (Castets et al., 2019), MAPK signaling pathway (Xue et al., 2016), hypoxia-inducible factor (HIF-1α) signaling pathway, p53 pathway, peroxisome proliferators-activated receptors (PPAR-γ) pathway, and nuclear factor kappa B (NF-κB) pathway (Sun et al., 2018; Hou et al., 2019). Generally speaking, the activation of AMPK-mTOR, MAPK, HIF-1α, and p53 are correlated with the upregulation of autophagy, while PI3K/Akt/mTOR, PPAR-γ, and NF-κB are relevant to the inhibition of autophagy. The formation of autophagy can be visualized by the expression level of autophagy modulators and markers, including Beclin1, LC3-phosphatidylethanolamine conjugates (LC3-II), P62, and lysosomal-membrane-associated proteins (Lamp) (Nabavi et al., 2019). Additionally, in recent years, the regulatory role of apoptosis-related proteins (Yang et al., 2015) and heat shock protein (HSP) (Zhan et al., 2017; Li et al., 2019a) in autophagy after cerebral ischemia has also gradually been recognized.

As mentioned before, available evidence suggests that autophagy plays a dual role in the fate of neurons and other cells in cerebral ischemia injury (Liu et al., 2020a). On the one hand, proper autophagy triggered by cerebral ischemia contributes to the clearance of damaged tissues and enable neuronal and other cells’ survival. The induction of autophagy in animal models confers the neuroprotection against cerebral ischemic injury (Wang et al., 2012; Jiang et al., 2015; Liu et al., 2019b). More specifically, autophagy removes damaged mitochondria through mitophagy, thereby reducing the generation of ROS (Nakka et al., 2016; Guan et al., 2018a; Guo et al., 2020). Moderate autophagy weakens neuroinflammation by inhibiting the activation of inflammasomes and regulating the phenotype alternation of microglia (Su et al., 2016; Mo et al., 2020). Additionally, autophagy-mediated endothelial cells and BBB protection have also been uncovered (Kim et al., 2018).

On the other hand, other investigators report that excessive autophagy activity complicates brain injury under ischemic conditions, while the inhibition of autophagy ameliorates cerebral ischemic injury (Liu et al., 2016; Zhang et al., 2019c). In one such study, AMPK-mediated autophagy enhanced oxidative stress and induced apoptosis in ischemic stroke models (Li and McCullough, 2010). Koike et al. found that mice deficient in autophagy induction gene Atg7 could be protected from hypoxic/ischemic-induced neuronal death (Koike et al., 2008). Another study showed that injection of the autophagy inhibitors 3-methyl-adenine (3-MA) and bafliomycin A1 (BFA) resulted in an inhibition on ischemia-induced upregulation of LC3-II (Wen et al., 2008). Moreover, two recent researches presented that inhibition of autophagy led to a decline in the level of oxidative stress following cerebral ischemic insult (Fu et al., 2019; Sun and Yue, 2019). Hence, the effect of autophagy on ischemic injured brain is a double-edged sword, which seems to rely on the balance between the amount of autophagy substrates and the clearance capability of autophagy. The regulation of autophagy has provided a therapeutic strategy for the intervention of ischemic stroke and achieved initial success (Nabavi et al., 2019).

**FIGURE 1:**
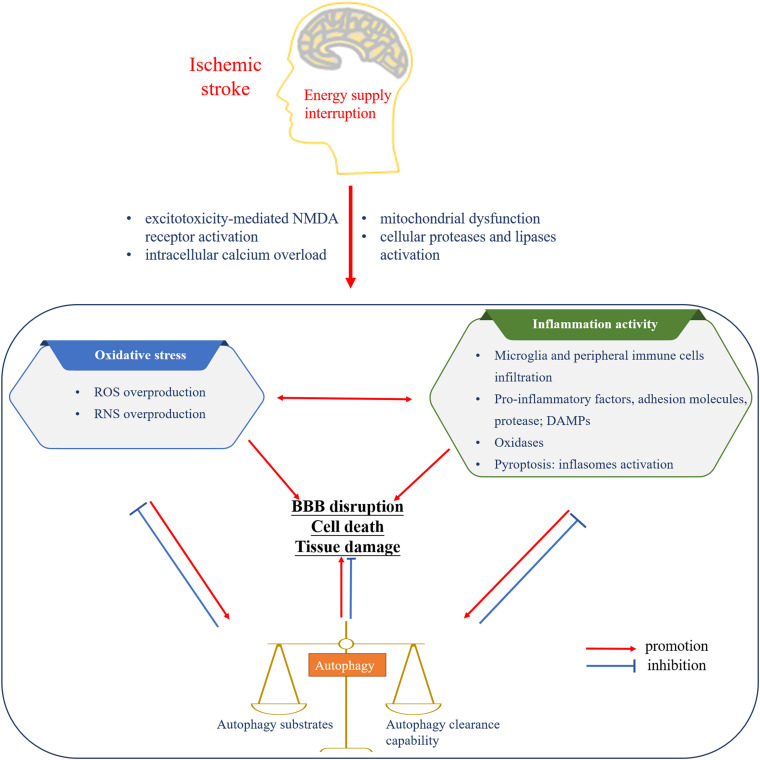
The interplay among oxidative stress, inflammatory activity, and autophagy following cerebral ischemia. The interruption of energy supply leads to oxidative stress, inflammatory activity, and autophagy. The mutual promotion between oxidative stress and inflammation induces the formation of autophagy and cerebral injury. Basic level of autophagy is beneficial to the inhibition of oxidative stress as well as inflammatory activity and brain recovery, while autophagy imbalance complicates the brain disaster. NMDA, N-methyl-D-aspartate; ROS, reactive oxygen species; RNS, reactive nitrogen species; DAMPs, dangerous-associated molecular patterns; BBB, blood–brain barrier.

Altogether, oxidative stress and inflammatory activity are reciprocal causation, advancing a cascade of injury responses following cerebral ischemia. Autophagy, triggered by oxidative stress and inflammatory activity, always in turn downregulate the level of oxidative stress and suppress inflammation responses. Meanwhile, relatively excessive and insufficient autophagy activities both aggravate the cerebral injury (Figure 1).

## Functionality and Therapeutic Efficacy of MSC-Based Therapy for Experimental Ischemic Stroke

### Introduction of MSCs and Secretome

Mesenchymal stem cells are defined as a type of adult stem cells that are capable of self-renewal and are culture expandable (Ferreira et al., 2018). MSCs can be obtained from the umbilical cord, bone marrow, dental pulp, adipose tissue, olfactory mucosa, and other tissues and have characteristics in common with mesenchymal tissue. MSCs possess multipotential differentiation ability, immunoregulatory properties, and low immunogenicity (Spees et al., 2016). The latter is due to its non-expression of major histocompatibility complex class II (MHC-II) and costimulatory molecules (Deans and Moseley, 2000), and these superiorities make allogeneic transplantation of MSCs possible.

In regenerative medicine, compared to the traditionally recognized cell replacement effect, the paracrine activities of MSCs, also known as MSCs secretome, is gradually attracting attention and considered to be more pronounced (Vizoso et al., 2017; L et al., 2019). This understanding is attributed to that MSCs secretome has the ability to cross the BBB, whereas merely 0.1–0.3% of intravenously transplanted MSCs is detected in the brain (Zagrean et al., 2018). In addition to the ability to pass the BBB, the advantages of MSCs secretome include no first-pass effect, lower risk of microvascular thrombosis, and the ease of producing large-scale genetically modified extracellular vesicles (EVs).

The secretome of MSCs includes soluble protein molecules and EVs. Soluble protein molecules are mainly composed of various chemokines, cytokines, and growth factors. EVs are divided into exosomes and microvesicles according to their different sizes, in which the diameter of exosomes is approximately 30–100 nm, while that of microvesicles is 50–1000 nm. The various molecules produced by MSCs are mainly involved in immune modulation and neuroprotection, and the latter is related to the neurogenesis and angiogenesis effects of neurotrophic factors and angiogenic factors. The EVs derived from MSCs mediate intercellular communication by RNAs and proteins (Li et al., 2020b).

In the past years, preclinical studies on the application of MSCs and secretome in ischemic stroke have increased significantly with inspiring outcomes (Cunningham et al., 2018). Next, we will discuss the relevant rationales of MSC and secretome to modulate oxidative stress, inflammation, and autophagy in experimental ischemic stroke building on the previous evidence.

### The Effect of MSCs on Oxidative Stress After Cerebral Ischemia

#### MSCs Inhibit the Production of ROS and RNS

Plenty of investigations have recapitulated that MSCs could rescue injured brain tissue via decreasing oxidative stress levels (Leu et al., 2010; Calio et al., 2014; Chen et al., 2016). Alhazzani et al. have concluded that MSCs coculture diminished the increased intracellular calcium and ROS of neuronal cells subjected to cerebral ischemia-like stress (Alhazzani et al., 2018). Li et al. (2012a) have found that adipose-derived mesenchymal stem cells (ADMSCs) attenuated cerebral ischemia–reperfusion injury through suppressing iNOS. Another two interesting findings indicated that olfactory mucosa mesenchymal stem cells (OM-MSCs) exert neuroprotective effects by rescuing the function of mitochondria and Golgi apparatus and enhancing antioxidation via upregulation of UBIAD1 and SPCA1 (He et al., 2020; Liu et al., 2020b). Moreover, that MSCs modified with diverse molecules exhibited antioxidative stress effects on ischemic stroke models have also been reported, such as C–C motif chemokine receptor 2 (CCR-2), brain-derived neurotrophic factor (BDNF), and circRNA-Akap7 (Lu et al., 2016; Huang et al., 2018b; Xu et al., 2020).

#### MSC-Mediated Mitochondrial Transfer

Recently, emerging evidence have suggested that MSCs can directly transfer healthy mitochondria to damaged cells and mitigate tissues damaged by mitochondrial dysfunction. The potential application prospects of MSC-mediated mitochondrial transfer in ischemic stroke have also been explored (Babenko et al., 2015). Prior preclinical stroke studies have noticed that MSCs could transfer healthy mitochondria to damaged neuron and astrocyte, and the observed transfer is dependent on cell-to-cell contact through the formation of tunneling nanotubes (TNTs) and Cx43-regulated gap junction, release of EVs, and cell fusion (Acquistapace et al., 2011; Lu et al., 2020). Additionally, the role of Miro1 in mitochondrial transfer has also been disclosed, where increasing Miro1 expression in MSCs improved the efficiency of mitochondrial transfer into the neurons and astrocytes (Babenko et al., 2018; Tseng et al., 2020). Generally, the replenishing of healthy mitochondria of the damaged neural cells is capable of enhancing oxidative phosphorylation, decreasing cellular oxidative stress level, and then alleviating the brain disaster cascade following cerebral ischemia (Pourmohammadi-Bejarpasi et al., 2020). Based on the foregoing discussion, MSCs exert antioxidant capacity in ischemic stroke mainly via reducing oxidative stress level and transferring healthy mitochondria.

Overall, researches regarding the alleviation effect of MSCs on oxidative stress following cerebral ischemia are not as common as those of other organs, perhaps due to the presence of the BBB restricting the arrival of systematically transplanted MSCs to cerebral ischemia lesions. For this reason, the MSCs secretome that can penetrate the BBB is peculiarly important. Massive anti-inflammatory molecules and neurotrophic factors secreted by MSCs directly lessen neuroinflammation and then reduce oxidative stress. Accordingly, we will expound in detail the underlying mechanisms of MSCs and secretome in inflammatory activity of ischemic stroke in the next section.

### The Role of MSCs in the Alleviation of Inflammatory Activity After Cerebral Ischemia

The anti-inflammatory mechanism of MSCs consists of immunomodulatory effect and EV-mediated microRNA (miRNA) molecular transfer. Besides, evidence about MSCs regulation of pyroptosis after cerebral ischemia have also been newly presented.

#### The Immunomodulatory Effect of MSCs and Neuroinflammation

The immunomodulatory effects of MSCs have been extensively studied in the peripheral regions. A variety of cytokines secreted by MSCs have been linked to their immunoregulatory function. These molecules include NO (in mice), IDO (in human), PGE2, TGF-β, HLA-G5, TSG-6, IL-1Ra, IL-10, and antagonistic variants of CCL2 (Wang et al., 2014). The production of these factors inhibits the differentiation, proliferation, and activation of various immune cell subgroups such as macrophages, neutrophils, T cells, B cells, natural killer (NK) cells, DCs, and mast cells, while it increases the generation of regulatory T cells (Treg) (Li and Hua, 2017). Moreover, coculture with MSCs promotes macrophages, DCs, T cells, and NK cells toward anti-inflammatory phenotypes *in vitro* (Cunningham et al., 2018). Interestingly, the immunomodulatory properties of MSCs depends on the type and intensities of inflammatory mediators present in the microenvironment. Diverse inflammatory states contribute to dramatically different responses to MSC therapy, suggesting a plasticity of MSCs in immunoregulation (Li et al., 2012b). For instance, MSCs are activated by proinflammatory signals through TLR3 and exert immunosuppressive effects, denoted as MSC2 phenotype, while TLR4 priming results in a proinflammatory phenotype (MSC1) accompanied by enhanced T cell responses in the absence of an inflammatory environment (Li et al., 2012b; Bernardo and Fibbe, 2013). It is worth mentioning that NO or IDO are key participants and might be the target of manipulating the plasticity of MSC-mediated immunomodulation.

In general, the anti-inflammatory molecules generated by MSCs also exhibit immunomodulatory properties in the central nervous system. The immunomodulatory and neuroprotective effects of MSCs in experimental ischemic stroke have also been gradually revealed. These beneficial effects are associated with the modulation of a number of processes, including elevated secretion of anti-inflammatory molecules accompanied by a decline in proinflammatory cytokines, inactivation of inflammatory cells, and inhibition of BBB leakage.

Early MSCs administration after stroke notably upregulated the expression level of IL-10, with decreased TNF-α level in the brain tissue (Liu et al., 2009; Boshuizen and Steinberg, 2018). Recent lines of evidence support MSCs regulation of immune cell infiltration and microglia/macrophage polarization in ischemic brain. Cheng et al. found that bone marrow mesenchymal stem cells (BMSCs) attenuated neutrophil infiltration, MMP-9 function, and BBB destruction by inhibiting the expression of intracellular adhesion molecule 1 (ICAM-1) in endothelial cells (Cheng et al., 2018). Another study suggested that MSCs therapy reduced astrocyte apoptosis and inhibited ischemia-induced aquaporin-4 (AQP4) upregulation, and this was related to the activation of p38 signaling pathway (Tang et al., 2014). Guan and his colleagues discovered that the overall number of Ly6C+ cells (monocytes/macrophages) in the infarct core was reduced by 50%, whereas the proportions of Ly6C+ cells coexpressing BDNF, IL-1β, or TNF-α were dramatically elevated following MSCs infusion (Guan et al., 2018b). Interestingly, the majority of Ly6C+ cells in the peri-infarct region was negative for BDNF, TNF-α, and IL-1β. This research suggested that Ly6C+ cells may consist of heterogeneous populations in the infarct area, which can be modulated by intravenously infused allogeneic MSCs. Meanwhile, that BMSCs inactivated the microglia and induced M2 polarization was further observed by other researchers (Li et al., 2019d; Yang et al., 2020c). Oh et al. (2018) demonstrated that human umbilical cord mesenchymal stem cells (hUMSCs) could relieve neuroinflammation in rodent stroke models by increasing interleukin-1 receptor antagonist (IL-1ra) expression in macrophage cell lines.

It has been recently shown that employed approaches *in vitro* were able to enhance the therapeutic potential of MSCs, like molecular priming and tissue engineering (Cunningham et al., 2018). Many researchers endowed MSCs with more potent anti-inflammatory properties using anti-inflammatory molecules. For instance, Cunningham and his colleagues’ work indicated that conditioned medium from IL-1α-primed MSCs contributed to improvements in behavioral outcomes (Cunningham et al., 2020). Other investigators found that CCL2-overexpressing hUMSCs or activation of MSCs with interferon-γ both induced a more forceful anti-inflammatory phenotype of MSCs relative to naive MSCs in ischemic stroke (Lee et al., 2020; Tobin et al., 2020). Another significative study showed that the presence of minority population of regulatory T cells in BMSCs conferred them with immunomodulatory and neuroprotection effects against stroke (Neal et al., 2019).

Furthermore, biomaterials have been reported to be able to modulate the behavior of stem cells. Zhai et al. demonstrated that nitrogen-doped carbon nanocages enhanced the therapeutic effects of hUMSCs on cerebral infarction and inhibited the microglia reactivation and neuroinflammation (Zhai et al., 2019). Besides, Huang and his group coated palmitic acid peptide onto the cell membrane of MSCs and thus increased the number of transplanted cells in the ischemic lesion (Huang et al., 2017). Generally, these approaches used to modify MSCs might generate a potential therapeutic strategy for stroke management.

#### MSC-Derived EV-Mediated miRNA Transfer and Neuroinflammation

That MSC-derived EVs can be advantageous over MSCs in the field of stroke therapy is partly dependent on the EV-mediated molecular transfer. EVs always serve as molecular cargoes, such as membrane receptors, proteins, lipids, and various forms of RNA molecules (Otero-Ortega et al., 2018). Among the contents of EVs, miRNAs, endogenously expressed RNA molecules that function to inhibit messenger RNA (mRNA) translation, have been shown to govern important processes that are responsible for ischemic stroke injuries (Khoshnam et al., 2017). Therefore, EV-mediated miRNA transfer provides an attractive candidate for the treatment of cerebral ischemic injury.

Recently, a number of studies have confirmed the therapeutic effectiveness of EV-mediated miRNA delivery in ischemic stroke. Plenty of miRNAs were involved in these processes, such as miRNA-29b-3p and miRNA-26b-5p (Hou et al., 2020; Li et al., 2020a). However, more researchers used miRNAs to modify MSCs for producing more robust EVs. In their investigations, EVs from MSCs primed with miRNA-17-92, miRNA-138-5p, miRNA-132-3p, miRNA-181b, and miRNA-223-3p showed stronger neuroprotection effects than EVs lacking additional miRNA. Those miRNAs mainly participated in the reduction in neuroinflammation, ROS production, as well as BBB dysfunction, and promotion of angiogenesis (Xin et al., 2017a; Yang et al., 2018; Deng et al., 2019; Pan et al., 2020; Zhao et al., 2020). Intriguingly, Xin et al. (2017b) proved that exosomes derived from miRNA-133b-overexpressed MSCs improved neural plasticity and functional recovery via stimulating the release of exosomes from oxygen and glucose deprivation (OGD)-treated astrocytes.

Moreover, other teams designed to modify MSCs in other ways to enhance the therapeutic potential of their EVs. A recent study showed that pretreatment of MSCs with lithium significantly upregulated the expression level of miRNA-1906 in MSC-derived EVs, thereby enhancing the resistance of cultured astrocytes, microglia, and neurons against hypoxic injury and reducing the levels of poststroke cerebral inflammation, and this process was connected with miRNA-1906 inhibition of TLR4 abundance (Haupt et al., 2020). Kim et al. (2020) found that magnetic extracellular nanovesicles derived from iron oxide nanoparticle (IONP)-harboring MSCs eminently promoted the anti-inflammatory response to cerebral ischemic injury, which they suggested was relevant to IONP stimulating the expression of therapeutic growth factors in MSCs. There was also a report on the effective inhibition of ROS and inflammatory activity following cerebral ischemia by combined nanoformulation of curcumin and embryonic stem-cell-derived exosomes (Kalani et al., 2016).

#### MSCs and Pyroptosis in Ischemic Stroke

Despite that studies on MSC-based therapies that target pyroptosis are relatively few in ischemic stroke, eminent outcome has also been observed. *In vitro*, the inhibitory effect of BMSC-derived exosomes on pyroptosis in PC12 cells was comparable to the NLRP3 inhibitor and was reversed by NLRP3 overexpression (Zeng et al., 2020). Meanwhile, that human umbilical cord blood mononuclear cells (cbMNCs) inhibited the activation of NLRP3 inflammasome *in vivo* has also been documented (Liu et al., 2018). Another *in vivo* investigation demonstrated that lymphocytes cocultured with human cord blood-derived multipotent stem cells (HCB-SCs) attenuated inflammasome activity in middle cerebral artery occlusion (MCAO) rats by suppressing NLRP3 inflammasome activation and promoting Tregs differentiation (Zhao et al., 2019c). In addition, a study on microglia revealed that hypoxia-preconditioned OM-MSCs suppressed pyroptotic death of microglia caused by cerebral ischemia–reperfusion insult by activating HIF-1α (Huang et al., 2020).

The mechanism by which MSCs and secretome inhibit pyroptosis has been more deeply studied in other disease models. Several new findings showed that MSCs exosomes inhibited NLRP3 expression and pyroptosis of cardiomyocytes and myocardial infarction by delivering miRNA-320b or long non-coding RNA (lncRNA) KLF3-AS1 (Mao et al., 2019; Tang et al., 2020). Liu et al. (2020c) found that cotransplantation of a chitosan thermosensitive hydrogel with BMSCs contributed to a better outcome in myocardial infarction, which is manifested by the inhibition of inflammatory response and alleviation of pyroptosis in vascular endothelial cells. There were also studies on MSC-derived miRNAs or circular RNAs (circRNAs) downregulating pyroptotic components in hypoxia/reoxygenation renal epithelial cells or ischemic muscle (Yuan et al., 2017; Yan et al., 2020). Besides, Kong and his group transplanted IL-37 gene-modified MSCs into rats model of intestinal ischemia–reperfusion injury and found that the expression of NLRP3 and downstream targets (cleaved caspase-1, IL-1β, and IL-18) were observably lessened (Kong et al., 2020). Overall, the underlying mechanism regarding multiple molecular pathways involved in the role of MSCs and secretome in other diseases are expected to further elucidate in ischemic stroke.

### The Role of MSCs in the Regulation of Autophagy Following Cerebral Ischemia

Different from the direct inhibition of oxidative stress level and inflammatory activity, MSCs have two-sided effects on autophagy in ischemic stroke.

#### MSCs Confer Neuroprotection Against Cerebral Ischemic Injury by Inhibiting Autophagy

Accumulating evidence have implied that MSCs were able to suppress autophagy through numerous molecular pathways and then promoted functional recovery after ischemic injury. First, the most studied was the ability of MSCs to activate the mTOR pathway, including PI3K/Akt/mTOR pathway (Gong et al., 2019; He et al., 2019; Li et al., 2019c; Liu et al., 2019a; Nazarinia et al., 2019; Lin et al., 2020) and PTEN/Akt/mTOR pathway (Li et al., 2020c). Among these researches, Li et al. (2019c) further found that silencing of SNHG12 in MSCs enhanced the effects of MSCs in reducing autophagy of OGD-treated brain microvascular endothelial cells (BMECs) and MCAO rats by activating the PI3K/AKT/mTOR signaling pathway. Another team’s results showed that SDF1 overexpression in MSC-derived exosomes also inhibited autophagy of ischemic myocardial cells through the same pathway (Gong et al., 2019). Second, exosome-mediated miRNAs delivery also took part in the regulatory process. miRNA-125b-5p and miRNA-25 delivered by transplanted MSC-derived exosomes inhibited p53/Bnip3-mediated autophagy (Woodall and Gustafsson, 2018; Kuang et al., 2020). miRNA-20a in exosomes from hUMSCs directly binds to beclin-1 and inhibits its expression, thereby inhibiting autophagic flux in ischemia–reperfusion-induced injury (Zhang et al., 2019d). Third, a recent study suggested that the protective role of transplanted MSCs in a murine model of ischemic stroke was associated with their promotion of the molecular switch from autophagy to ubiquitin–proteasome system (UPS) (Tadokoro et al., 2020).

#### MSCs Exhibit Neuroprotection in Ischemic Stroke by Enhancing Autophagy

By contrast, some other investigations declared that MSCs combated ischemic injury by enhancing autophagy (Huang et al., 2018a; Yang et al., 2020a). Likewise, in most studies, MSCs play a role by targeting mTOR-mediated autophagy pathway. In PC12 cells treated with OGD insult, BMSC exosomes attenuated the pyroptosis mediated by NLRP3 inflammasome by promoting AMPK-dependent autophagy flux (Zeng et al., 2020). Other data demonstrated that BDNF/mTOR signaling pathway, PI3K/Akt/mTOR pathway, and Notch2/mTOR pathway were also involved in the regulation process of MSCs promoting autophagy (Li et al., 2015a, 2019b; Zheng et al., 2018). Besides, heme oxygenase-1 (HO-1)-mediated autophagy could also be modulated by MSCs in ischemic injury models (Wang et al., 2018c).

Collectively, the regulatory role of MSCs in autophagy following ischemic stroke is still under dispute. Even in the same cell or animal models of cerebral ischemic injury, MSCs can exhibit diametrically opposite effects on the modulation of autophagy, which is believed to be related to multiple factors, such as the length of modeling time and the time nodes of MSCs intervention. From another point of view, the beneficial or detrimental impacts on ischemic brain tissue depend on the intensity of autophagy, and the transplanted MSCs exert neuroprotection effects through modulating their functions adaptively according to the state of autophagy.

## Conclusion and Perspective

**FIGURE 2:**
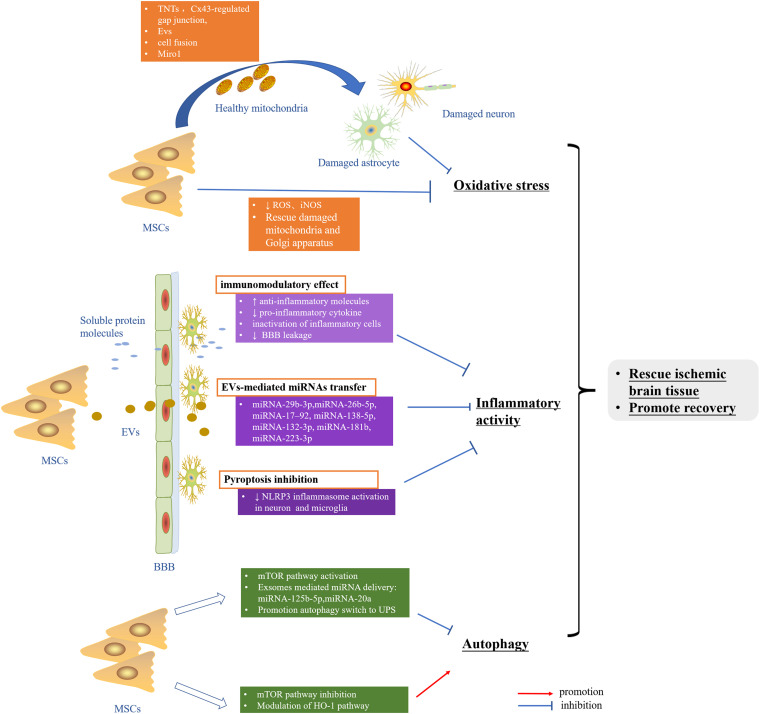
MSCs rescue ischemic brain tissue and promote recovery by inhibiting oxidative stress as well as inflammatory activity and modulation autophagy. MSCs, mesenchymal stem cells; TNTs, tunneling nanotubes; EVs, extracellular vesicles; ROS, reactive oxygen species; RNS, reactive nitrogen species; BBB, blood–brain barrier; UPS, ubiquitin-proteasome system; HO-1, heme oxygenase-1.

The applicable therapeutic strategy to reduce or prevent the cerebral ischemic injury is still largely lacking. Abundant data implicated intricate rather than a single signaling pathway to frequently work together to undermine the cells in the setting of cerebral ischemia–reperfusion. The crosstalk among oxidative stress, inflammatory activity, and autophagy dysfunction may raise the need of deeply taking into consideration these pathways in ischemic stroke. Nowadays, the pleiotropic ability of MSCs to exhibit antioxidative stress, reduce neuroinflammation, and regulate autophagy in experimental ischemic stroke has been recognized, most of which benefit from its robust paracrine activities (Figure 2). More importantly, the low immunogenicity, ability to cross the BBB, capacity of targeted delivering gene drugs, and similar properties as MSCs seem to make MSC-derived EVs a better clinical application candidate relative to MSCs. In summary, MSCs and secretome hold great promise in the clinical treatment of ischemic stroke.

## Author Contributions

ZH and HX acquired the funding. JH attended in literature review and drafting the manuscript. JL and YH participated in literature review. XT and ZH supervised the project. All authors read and approved the final manuscript.

## Conflict of Interest

The authors declare that the research was conducted in the absence of any commercial or financial relationships that could be construed as a potential conflict of interest.

## Abbreviations

MSC: mesenchymal stem cells
BBB: blood–brain barrier
ROS: reactive oxygen species
RNS: reactive nitrogen species
O_2_^–^: superoxide anions
NO: nitric oxide
ONOO^–^: peroxynitrite anion
MRC: mitochondrial respiratory chain
NMDA: N-methyl -D-aspartate
NOS: nitric oxide synthase
DCs: dendritic cells
TNF- α: tumor necrosis factor- α
IL-1: interleukin-1
iNOS: inducible nitric oxidase synthase
IFN: interferon
TLR4: Toll-like receptor 4
DAMPs: dangerous-associated molecular patterns
HMGB1: high-mobility group box 1 protein
CMA: chaperone-mediated autophagy
mTORC1: mammalian target of rapamycin complex 1
AMPK: AMP-activated protein kinase
MAPK: mitogen activated protein kinase
HIF-1 α: hypoxia-inducible factor
PPAR- γ: peroxisome proliferators-activated receptors
NF- κ B: nuclear factor kappa B
LC3-II: LC3-phosphatidylethanolamine conjugates
LAMP: lysosomal membrane-associated proteins
HSP: heat shock protein
MHC-II: major histocompatibility complex class II
EVs: extracellular vesicles
ADMSCs: adipose-derived mesenchymal stem cells
OM-MSCs: olfactory mucosa mesenchymal stem cells
CCR-2: C–C motif chemokine receptor 2
BDNF: brain-derived neurotrophic factor
TNTs: tunneling nanotubes
NK: natural killer
Treg: regulatory T cells
BMSCs: bone marrow mesenchymal stem cells
AQP4: aquaporin-4
hUMSCs: human umbilical cord mesenchymal stem cells
OGD: oxygen and glucose deprivation
IONP: iron oxide nanoparticles
cbMSCs: cord blood mononuclear cells
HCB-SCs: human cord blood-derived multipotent stem cells
MCAO: middle cerebral artery occlusion
UPS: ubiquitin-proteasome system
HO-1: heme oxygenase-1.
